# The role of need for cognition and perceived self-efficacy in modulating emotional and behavioural responses to failure

**DOI:** 10.1186/s40359-025-03383-8

**Published:** 2025-09-17

**Authors:** Michaela A. Reuter, Sören Enge, Monika Fleischhauer

**Affiliations:** Department of Psychology, Faculty of Natural Sciences, MSB Medical School Berlin, Berlin, Germany

**Keywords:** Need for cognition, Perceived self-efficacy, Failure, Anger, Persistence

## Abstract

**Background:**

Need for cognition (NFC) measures the enjoyment of and the search for intellectual challenges and is probably the best-known construct for measuring cognitive motivation. Several studies have shown that individuals high in NFC invest more mental effort in complex tasks, process information more deeply and perceive themselves to be effective problem solvers. To date, however, there has been little research investigating the need for cognition in relation of failure in cognitively challenging tasks. Therefore, in this study, we investigated whether NFC influences the extent to which anger is experienced after a failure in cognitively demanding tasks and whether individuals high in NFC are more intended to continue after failure (persistence) than those low in NFC.

**Method:**

A total of 194 participants took part in this study. Each participant completed two solvable tasks (one anagram, one tangram; control-condition) and two unsolvable tasks (one anagram, one tangram) to generate the experience of failure. After completing each task, the participants were asked to report their state anger. Furthermore, after each of the two unsolvable tasks, the participants had to decide whether they wanted to solve the tasks again to assess their intention to persist after a failure. Based on studies showing that perceived self-efficacy (PSE) influences emotional responses to failure, PSE was also considered as an independent variable and as a moderator of the relationship between NFC and the experience of failure.

**Results:**

The results demonstrate that NFC positively affects feelings of state anger after failure in cognitively challenging tasks and that this effect was moderated by PSE. Furthermore, state anger positively affected the intention to show persistence in individuals higher in NFC and PSE.

**Discussion:**

Thus, these experimental results support conceptual assumptions of NFC as intrinsic motivation to engage in cognitive endeavors and underline the potentially important role of NFC and PSE in modulating responses to failure.

## Background

Most individuals will face obstacles, when trying to achieve personal goals. Those who expend large amounts of motivation but are still not able to achieve their goal will most likely be frustrated and hence, experience failure [1]. Thereby, failure can be defined as non-attainment of a desired goal [2–5]. By that, failure may result from a negative evaluation of whether the results of goal-oriented behavior match an individual’s expectations or not [5]. Since striving for goals is an affective experience [6] and failure is an event of frustration [1, 2], negative emotions may arise. One main emotional response that arises from the blocking of a desired goal [7, 8] and that typically occurs after failure [1, 2, 9–11] is anger.

Because personality traits have an impact on task engagement and motivation [12], responses to failure might be related to individual differences in personality. For example, Besser et al. [13] showed that self-oriented perfectionism (i.e. high personal standards) is a vulnerability factor in this context. After receiving negative performance feedback, individuals with self-oriented perfectionism experienced a significant increase in negative affect and a decrease in positive affect accompanied by distress-related cognitive reactions such as rumination about mistakes. Moreover, neuroticism has been often associated with emotional responses to negative events. For example, Robinson et al. [14] examined the role of neuroticism in shaping responses to negative feedback, revealing that individuals with low levels of neuroticism tend to improve accuracy by slowing down after error feedback, whereas those with high neuroticism exhibit reduced accuracy under similar conditions. These findings suggest that personality traits such as neuroticism and self-oriented perfectionism may be critical factors in distinguishing between functional and dysfunctional mechanisms in behavioral adjustments following negative feedback.

In addition to these vulnerability factors, it might be valuable to consider personality traits that may lead individuals to cope with failure more adaptively. By that, also individuals who are more motivated and engaged might show stronger emotional responses but more adaptive behaviour in the event of failure [1, 15]. One personality construct that could be of particular interest in this context is the Need for Cognition (NFC).

### Need for cognition and response to failure

NFC is defined as the “intrinsic motivation to engage in and enjoy effortful cognitive endeavors” [16, p. 197]]. Individuals high in NFC “perceive themselves […] to be effective problem solvers […]” [16, p. 243], process information more deeply, and invest more mental effort in complex tasks [17]. Petričević et al. [18] observed that NFC positively correlates with the wish to develop or demonstrate competency. Moreover, Steinhart and Wyer [19] reported that individuals high in NFC showed greater motivation to avoid failure and to avert an incompetent self-image. Given these findings solving a cognitively effortful task is most likely more relevant to individuals higher than to those lower in NFC. Therefore, the emotional response to failure in a cognitively challenging situation may be stronger for individuals high in NFC, particularly in light of the positive association between task motivation and frustration following failure [1]. In contrast, individuals lower in NFC generally show little motivation in cognitively challenging tasks as neither the possibility of failure nor the possibility of success in such tasks seem to motivate them [19]. As a result, low-NFC individuals may experience less anger after failure.

### The moderating role of perceived Self-Efficacy

The effort invested in solving cognitively demanding tasks can also be linked to Perceived Self-Efficacy (PSE) [5, 20–23], which can be described as an expectation of one’s own competence and confidence in being able to cope with a situation using one’s own abilities [22, 24]. PSE is formed by attributing experiences of failure and success to individual actions [20]. Thereby, PSE influences emotional reactions [21] as well as persistence and performance goals [5, 20, 25].

Self-efficacy has been shown to be positively related to NFC [26–30] and to mediate the relationship between NFC and performance outcomes such as academic performance [27] or performance in highly cognitively demanding tasks [31]. Thereby, individuals with higher NFC reflect events more thoroughly and integrate them in their sense of self [32]. In line with that, Dickhäuser and Reinhard [33] reported that the individual’s self-concept of being able to solve a specific cognitively demanding task affected performance expectancies stronger when NFC was high, indicating that NFC and PSE may interact in the emotional response to failure.

Because the experience of failure follows from the evaluation of results based on expectations [5], it can be presumed that PSE, and with that the expectation about one’s own competence, influences the effect of NFC on anger after failure. Thus, we hypothesize that the positive relationship between NFC and anger after failure might be moderated by PSE. By that, we presume that the effect of NFC on anger after failure will be stronger in individuals higher than in those lower in PSE and furthermore, that individuals with a higher NFC will show more anger in response to a failure in a cognitively demanding situation the higher their expectations of their abilities (PSE) is. In contrast, individuals with lower NFC might respond with lower anger relatively independent of their PSE. In line with that, Osberg [34] showed that low NFC-individuals were hardly affected by expectations of their performance. Hence, in this study we further want to address whether PSE moderates the effect of NFC on anger and moreover, whether possible effects of NFC on anger after failure are specific for NFC, while controlling for shared variance with PSE.

### Behavioral responses to failure

After a failure, individuals tend to show behavioral responses [9, 35, 36] to reduce aversive emotional states [9, 36]. For example, individuals may show persistence of action or leave the situation [9, 37]. Emotions such as anger play an important role here [6, 10, 11, 38]. Weber [36] for example showed that anger functions as impetus for further action. Angry individuals may consider future actions, formulate plans, are more likely to execute planned behavior and are eager to address the cause of anger [4, 39]. If there is an opportunity to resolve the anger-producing event, anger can be considered an activating affective state that can be associated with approach motivation and the feeling of being in control [4, 7, 8, 40]. Schmitt et al. [40] reported that anger might lead to greater persistence especially when importance and complexity of a goal are rather high and individuals are committed. Thus, anger could positively affect the intention to continue (i.e. being persistent) after failure.

Aside from anger, NFC and PSE might positively affect the intention to show persistence after failure. NFC positively correlates with conscientiousness [41, 42], achievement motivation [43], the motivation to approach and overcome challenges [28], and the investment of behavioral and cognitive effort [18, 44, 45]. Individuals higher in NFC describe themselves as more goal-orientated, self-controlled and more persistent than those low in NFC [22, 41, 46–48]. Moreover, NFC correlates positively with the believe in greater resources and with willpower [49] while it correlates negatively with demand avoidance [45].

Similarly, PSE is associated with the amount of effort spend on solving tasks [20], with greater performance goals [25], greater motivation to complete a task [5], and less fear of failure [24]. Thereby, expectations in regard to PSE “contain a motivational component that determines when and for how long one will engage in overt behaviors to produce a desired outcome” [28, p. 361]]. In the event of failure, individuals with greater PSE attribute this failure to skills that are still achievable and to spending not enough effort instead of attributing it to immutable factors [21].

Based on these findings, we hypothesize that both NFC and PSE positively affect the intention to persist after failure in a cognitively demanding task. However, due to the overlap between NFC and PSE-related constructs such as self-esteem, self-worth and feeling in control [32, 34, 43, 48], it will be necessary to examine, whether NFC and PSE can independently explain variance in persistence behavior. Additionally, we will explore whether and how NFC and PSE interact with anger as an activating emotion.

### Present research

The main aim of this study is to examine the influence of NFC on responses to failure in a cognitively demanding task. We expect a positive association between NFC and anger after failure. Furthermore, we hypothesize that PSE will moderate the effect of NFC on anger, with stronger effects of NFC on anger after failure expected in individuals with higher PSE. Anger, as an activating emotion, may further positively influence the intention to persist after failure. Given, the above reported correlates of NFC and PSE with goal-directed and persistent behavior, we also expect positive direct effects of both traits on the intention to persist. Additionally, we will investigate whether an interaction between NFC, PSE, and anger influences the intention to persist after failure. Specifically, we expect that anger following failure will be more strongly related to the intention to persist in individuals with higher NFC and PSE.

## Methods

### Participants

The sample was recruited by sharing an online link to the survey via e-mail (private mailing and contacts list of the study’s director), on messenger services (WhatsApp) and on social media (profile of the study’s director on facebook and instagram). Further, the link could also be forwarded by the participants.

The link was clicked on 1074 times. Two-hundred-ten individuals completed the study. One individual stated to not have understood the instruction and hence, was excluded from the sample. Fifteen participants did not solve the two solvable tasks, which were so simple that they could have been solved in the time available (3 min). Therefore, these individuals were excluded from the sample, as their failure to complete these simple tasks may have suggested that they either did not understand the instructions or were not sufficiently engaged, both of which could have compromised the validity of the results. All participants had to be older than eighteen years of age. There were no other exclusion criteria. Thus, the final sample included a total of *N* = 194 participants (57% female; mean age = 36.4, *Mdn* = 28, *SD* = 15.8, range: 18–75 years).

### Procedure

The study was conducted in accordance with the Declaration of Helsinki (revised version) and the ethical standards of the German Psychological Association. The ethics committee of the MSB Medical School Berlin evaluated the study procedure and declared it to be ethically unobjectionable (committee’s reference number: MSB-2024/162).

An online survey (accessible for approximately four weeks in February and March 2020) was administered via UniPark (Questback GmbH, 2017). At the beginning of the survey, each participant was informed about the purpose of data collection and data security. The participants were informed that participation in study was completely voluntary and that the data would be collected anonymously. Anonymous data collection was ensured by the fact that the participants assigned themselves a code that only they could associate with their person. Participants were also informed that they could withdraw from participation at any time without any consequences. All participants gave their informed consent by pressing a confirmation button at the beginning of the survey, confirming that they were over eighteen years old and that they had been informed about the study conditions.

**Fig. 1:**
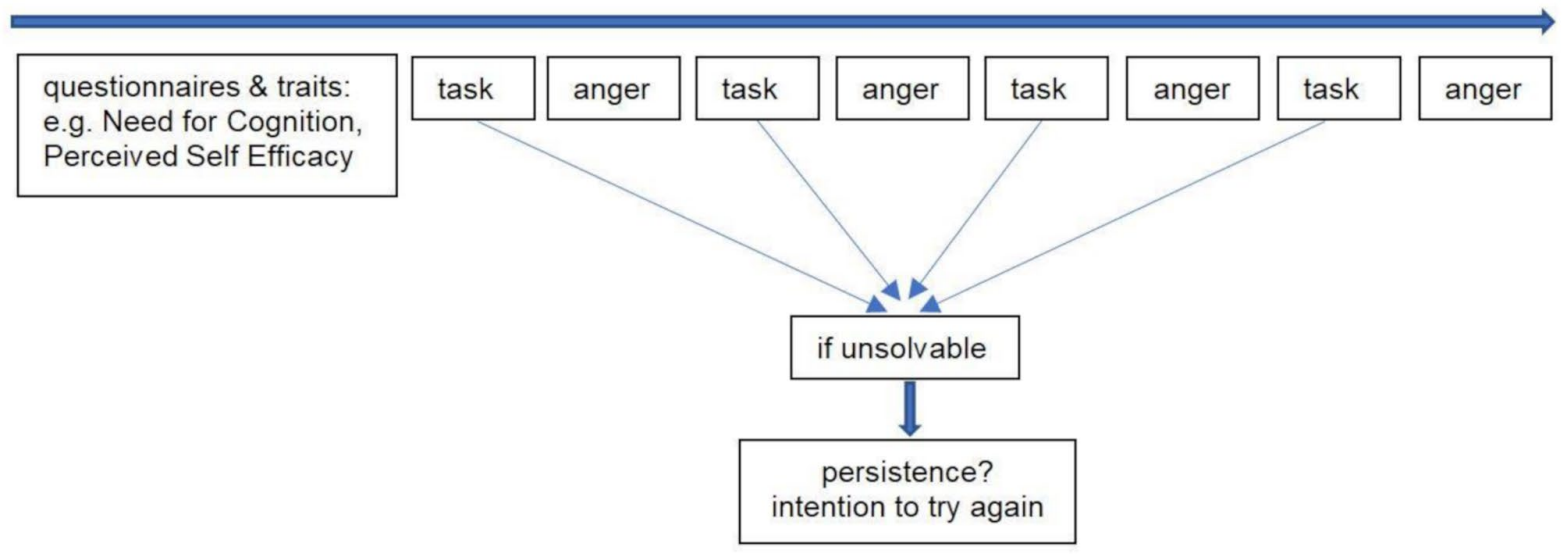
Method of collecting data for each participant Note. Each participant works on four different tasks - two anagram and two tangram tasks. After each task, state anger is assessed with the STAXI [52] questionnaire. One anagram and one tangram task are unsolvable. Only after those two tasks, persistence will be assessed. The order of tasks is randomly assigned

We used an experimental within-subject design to create a cognitively demanding situation and to systematically induce task failure within such a context. After participants completed several questionnaires (among others, measuring NFC, PSE, and baseline state anger), they were asked to work on four cognitive tasks - two tangrams and two anagram tasks. Each participant completed two solvable tasks (one anagram, one tangram; control-condition) and two unsolvable tasks (one anagram, one tangram; failure condition). All tasks were constructed to require focused cognitive effort and engagement. The two solvable tasks were designed to be moderately challenging but achievable within the given time frame, while the unsolvable tasks were intentionally constructed to appear solvable – thereby maintaining high cognitive demands – yet could not be solved, regardless of effort. To achieve this without raising participants’ suspicion, the unsolvable tasks were designed to be more complex (e.g., more puzzle pieces or longer letter combinations), so that their unresolvability would be perceived as high difficulty rather than as impossibility.The type of task (anagram or tangram) to work on first and whether participants received a solvable or unsolvable task first, was randomly assigned to control for order effects. The procedure is illustrated in Fig. 1. Each task had a maximum duration of 3 min. If participants did not solve the task within this time frame, it was automatically terminated. After each task, a questionnaire measuring anger was presented. Following the two unsolvable tasks, an additional measure of intention to persist was administered. To ensure data completeness, participants were required to answer all items before proceeding. Prior to the main study, we conducted a small pretest with three individuals to confirm that all tasks were perceived as cognitively effortful and that the unresolvability of the failure-condition tasks was not obvious.

Participants were informed that they could contact us at any time to learn more about the studies purpose and results. There was no reimbursement for participation in the study. The total processing time was about 30 min. The study was not preregistered. We report all manipulations, measures, and exclusions.

### Measures and material

NFC was measured with the German adaption of the 16 item NFC-Scale [50] (e.g. “ I really enjoy a task that involves coming up with new solutions to problems.”). Responses are recorded on a 7-point-scale ranging from 1 (completely disagree) to 7 (completely agree). In the present study scores varied between 2.7 and 5.9 (*M =* 4.7, *SD* = 0.8). The internal consistency of the scale was *α* = 0.90.

PSE was assessed with the 10-item German version of the General Self-Efficacy Scale [51] (e.g. “When I am confronted with a problem, I can usually find several solutions.”). Responses are recorded on a 4-point-scale ranging from 1 (not at all true) to 4 (exactly true). Item-scores are added up to a sum-score between 10 and 40. Scores varied between 13 and 40 (*M* = 29.8, *SD* = 4.5). The scale showed an internal consistency of *α* = 0.90.

State anger was assessed using the 10-item state anger scale and trait anger was assessed using the 10-item trait anger scale of the German adaption of the State-Trait-Anger-Inventory [52]. Responses are recorded on a scale from 0 (not at all) to 3 (very much so). Internal consistency for the state-scale was *α* = 0.91 − 0.96 for different measurement points. Trait anger scores varied between 0.1 and 2.2 (*M* = 0.9, *SD* = 0.4). The trait-scale’s internal consistency was *α* = 0.77.

The intention to persist was measured after working on an unsolvable task using a single, study-specific item: “Do you want to try solving the task again after completing the questionnaire?”. Individuals could respond with “yes” or “no”. A “yes” response was interpreted as indicating an intention to persist, whereas a “no” response indicated no such intention. To capture persistence in this specific task setting, we developed a context-sensitive item directly reflecting the immediate decision participants faced after task failure. Single-item measures are increasingly recognized as valid tools for assessing clearly defined psychological constructs, especially when minimizing participant burden is important [53, 54]. Given the overall survey duration (∼ 30 min), we opted for this concise and straightforward measure to reduce participant fatigue and dropout risk.

Moreover, to avoid frustration related to the survey’s length, which could interfere with participants’ emotional responses to failure, we chose to measure intention rather than actual persistence behavior. Intention can be defined as decision to perform particular actions [55]. Earlier research has shown that persistence can be assessed by using questionnaires [56–59]. Meier and Albrecht [60] proposed a model of persistence in which behavior follows intention and stronger intentions lead to greater persistence. Thereby, persistence consists of three stages: (1) defining a goal and willingness to establish it in response to failure, (2) deciding how to establish this goal, (3) deciding whether the outcome is acceptable; determining how long persistence occurs [60]. The question used in this study covers the first and second stages by asking, whether participants want to try again (stage 1) and also including information about action and time (stage 2). Meier and Albrecht [60] reported that personality variables (e.g. motivation, self-efficacy) may affect the implementation of action primarily at stage two. The third stage was of less interest and hence, actual behavior and its duration was not necessary to be assessed. Moreover, previous research has shown that implementation-intentions that specify behavior and situation of goal attainment make it more likely to actually act as intended even after encountering barriers [55, 61].

Moreover, data on gender, age, and level of education were collected. We assessed further questionnaires in the beginning of the survey that however, are not relevant for the present research questions and therefore are only mentioned at this point but are not further described. Thereby, we additionally conducted the Münchner Persönlichkeitstest [62], the Action Regulating Emotion Systems Scales [63], the Positive and Negative Affect Schedule (PANAS) [64], a single-item scale to assess willingness to take risks (R-1) [65], and a short scale to assess internal and external locus of control (IE-4) [66].

### Data analysis

All analyses were conducted using IBM SPSS Statistics 28 (SPSS Inc., Chicago, IL, USA). First, to test whether anger can be considered an emotional response to failure (within subject factor: before task, after solvable task, after unsolvable task) and to analyze effects of NFC on anger we calculated two mixed models one for each type of task (anagram and tangram). We entered NFC, age, trait anger, and PSE as covariates and gender as between-subject factor to measure the incremental value of NFC over and above other potentially relevant variables. Due to quite similar results in both task models (Table 1) we calculated average anger-scores for both types of tasks (mean scores: *working on a solvable task; working on an unsolvable task*). Hence, results for mean anger-scores are reported and were used in all further calculations. Second, it was assessed whether PSE moderates the effect of NFC on anger after failure by calculating a moderation analysis using PROCESS [67]. Thereby, we used a moderation model including NFC as independent variable, PSE as moderator, and anger after failure as dependent variable. Third, to assess whether anger after failure affects intention to persist, a multinomial logistic regression model was conducted. We used persistence as categorical variable with the values “0” = “not wanting to try again at all” (*n* = 62) and “1” = “wanting to try again after only one task” (n = 46) and “2” = wanting to try again after both tasks” (*n* = 86). NFC, PSE, anger and interactions of anger with the two personality traits were included as covariates.

Residuals of anger at different points of measurement were not distributed normally. However, outliers were not removed from the dataset because neither did exclusion lead to a normal distribution of residuals nor did the results change substantially. To control for normal distribution of residuals we used bootstrapping in the logistic regression and moderation analyses. Moreover, White-Test showed that there was homoscedasticity in anger at baseline (*χ²*(19) = 17.54, *p* = .553) and after success (*χ²*(19) = 12.54, *p* = .861) but heteroscedasticity in anger after failure (*χ²*(19) = 34.45, *p* = .016). However, Tabachnik and Fidell [68] demonstrated that valid analyses can be performed even if residuals are heteroscedastic. Hayes and Cai [69] suggested using HC4 in SPSS^®^ Statistics by IBM for robust and heteroscedasticity-consistent standard errors. Comparing parameter estimation in the mixed model with robust standard deviation (HC4) to the mixed model calculated before, there were no substantial differences. Greenhouse-Geisser correction procedure was used in the mixed models when sphericity was violated.

The datasets generated and analyzed in the current study, the syntax, and the dataset legend as well as the tangram tasks are available in the figshare repository, DOI’s: 10.6084/m9.figshare.14892606, 10.6084/m9.figshare.14892816, 10.6084/m9.figshare.14892786, and 10.6084/m9.figshare.29365304.v1. Anagrams used were *negrom* (solved: Morgen) and *reknkeusnha* (unsolvable).

**Table 1:** Means and standard deviations of state anger-Scores in each experimental condition

Condition	M	SD	Range	Cronbachs α
Baseline^a^	1.3	3.1	0–27	0.92
Anagram				
Success^b^	0.8	2.5	0 – 22	0.92
Failure^c^	3.9	5.8	0 – 22	0.95
Tangram				
Success^b^	0.9	2.8	0 – 20	0.91
Failure^c^	4.5	6.5	0 – 30	0.96
Mean-Scores^d^				
Success^b^	0.9	2.5	0 – 20.5	
Failure^c^	4.2	6.0	0 – 25	

*Note. N* = 194. ^a^before working on a task. ^b^after working on solvable tasks. ^c^after working on unsolvable tasks. ^d^mean scores of anger after working on the anagram and tangram tasks were used

### Power analysis

We performed a sensitivity power analysis in G*Power 3.1 for a repeated measures ANCOVA (within-between interaction), assuming alpha = 0.05. A sample of 194 would provide 80% power to detect an effect of Cohen’s *f* = 0.08. A sensitivity power analysis for moderation analysis using the power-analyses for a linear multiple regression (fixed model, single regression coefficient, two tailed) [70] showed that a sample of 194 would provide 80% power to detect an effect of Cohen’s *f* = 0.04. For assessing power for the logistic regression analysis, we conducted a Monte Carlo simulation in Anaconda (Anaconda Inc., Austin, TX, USA) using Python. Using 10,000 simulation runs, the simulation showed a power of 100% to observe the expected effects of PSE and NFC on anger, a power of 99% to observe an effect of anger*PSE and a power of 96% to observe an effect of the interaction anger*PSE*NFC.

## Results

### Descriptives for differences in anger scores among conditions

Descriptive analysis indicate that participants showed higher anger after failure (unsolvable task). Also, variance of anger scores was greater after failure (Table 2). At baseline 58% (*n* = 113), after success 67% (*n* = 130), and after failure only 27% (*n* = 52) of participants did not show any anger. Further, Table 2 shows that correlation coefficients did not exceed 0.7 and hence, indicates the absence of multicollinearity.

**Table 2:** Descriptive statistics and intercorrelations of study variables

Variable	M	SD	1	2	3	4	5	6	7	8
1. Anger baseline	1.3	3.1								
2. Anger success	0.9	2.5	0.63^***^							
3. Anger failure	4.2	6.0	0.25^***^	0.33^***^						
4. NFC	4.7	0.8	− 0.14^*^	− 0.08	0.13					
5. PSE	29.8	4.5	− 0.28^***^	− 0.28^***^	− 0.01	0.42^***^				
6. Trait Anger	0.9	0.4	0.13	− 0.09	0.40^**^	− 0.20^**^	− 0.23^**^			
7. Age	36.3	15.8	− 0.02	− 0.09	0.40^**^	− 0.02	0.18^*^	0.16^*^		
8. Gender			0.10	0.04	0.12	− 0.11	− 0.19^**^	− 0.03	0.02	

*Note*. *N* = 194. Pearson correlation two-tailed. Mean scores of anger after working on the anagram and tangram tasks were used

^*^p < .05. ^**^p < .01. ^***^p < .001

### Anger after failure

We applied a mixed model including anger (at baseline, after success, and after failure) as repeated factor and NFC as covariate. Further, to consider potentially confounding factors and to show the incremental value of NFC, we added age, PSE, and trait anger as covariates and gender as a between subject factor (variables associated with state anger and/or with NFC; see Table 2). The analyses showed a main effect of point of anger measurement (*F*(1.39, 261.55) = 12.45, *p* < .001, partial *η2* = 0.062) with anger at baseline (*M =* 1.2, *SD* = 0.2, 95% CI [0.8, 1.7]), after success (*M =* 0.8, *SD* = 0.2, 95% CI [0.5, 1.2]), and after failure (*M =* 4.0, *SD* = 0.4, 95% CI [3.3, 4.7]). Thereby, Bonferroni adjusted post-hoc analysis showed an increase in anger between anger at baseline and anger after failure (2.8, 95% CI [1.9, 3.7], *p* < .001) as well as between anger after success and anger after failure (3.2, 95% CI [2.4, 4.0], *p* < .001) but no difference between anger at baseline and anger after success (-0.4, 95% CI [-0.8, 0.0], *p* = .089).

### The effect of NFC on anger

**Fig. 2:**
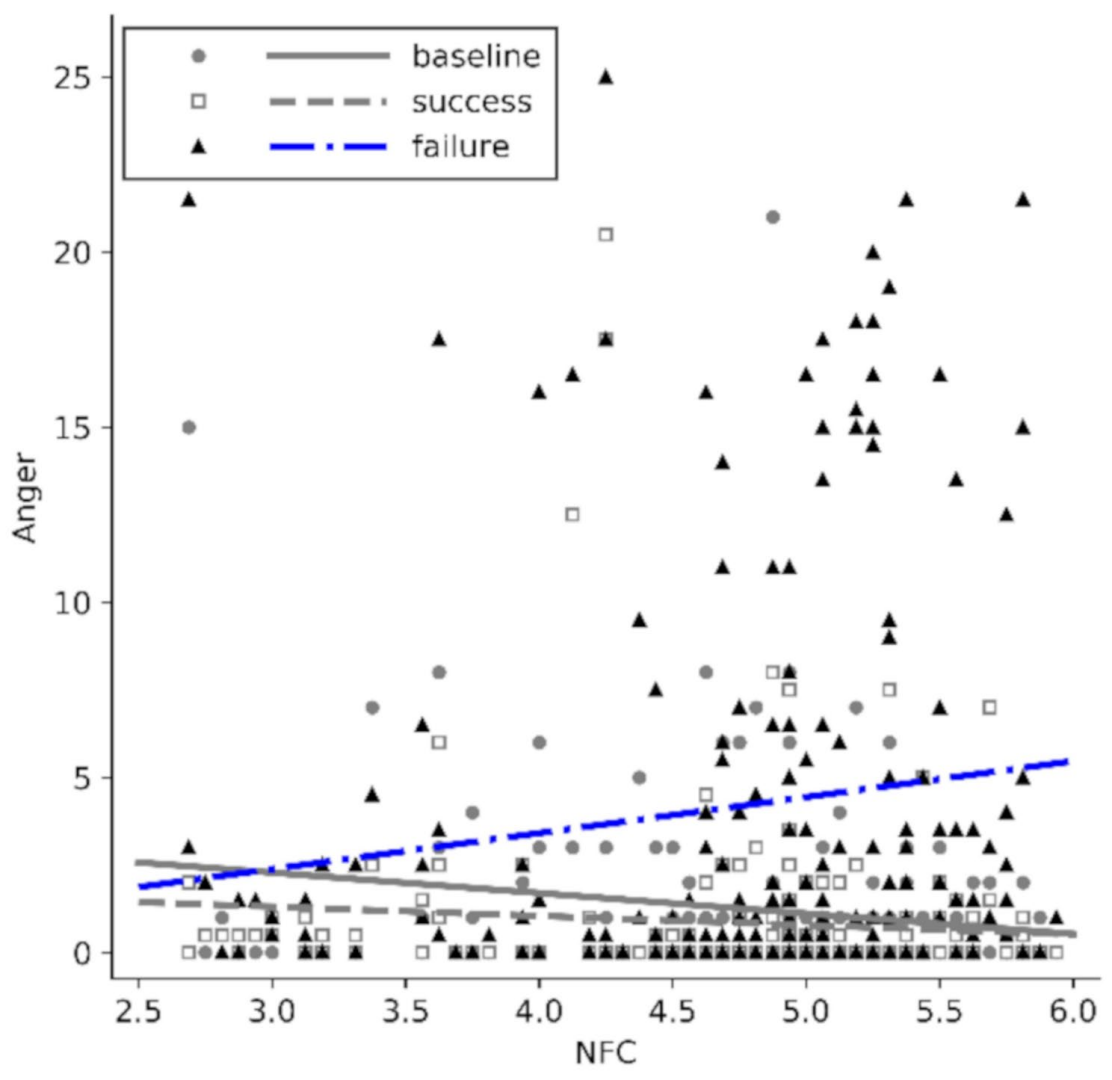
Regressions-slopes of NFC on anger at baseline, after success and after failure Note. *N* = 194. Mean scores of anger after working on the anagram and tangram tasks were used

Furthermore, the mixed model revealed an interaction effect of NFC × point of anger measurement (*F*(1.39, 261.55) = 14.76, *p* < .001, partial *η2* = 0.073). The effect size of the interaction was medium *(f*_*Cohen*_ = 0.28). A graphical analysis of regression-slopes suggested heterogeneity of slopes at different points of measurement. In contrast to anger after success or at baseline, for anger after failure the regression-slope of NFC was positive (Fig. 2). Parameter estimation revealed no effect of NFC on anger at baseline (*b* = − 0.09, 95% CI [-0.71, 0.53], *t* = − 0.28, *p* = .778, partial *η2* = 0.00) or after success (*b* = 0.13, 95% CI [-0.36, 0.63], *t* = 0.52, *p* = .604, partial *η2* = 0.00), but did show a positive effect of NFC on anger after failure (*b* = 2.04, 95% CI [1.03, 3.05], *t* = 3.98, *p* < .001, partial *η2* = 0.08). The interaction effects of NFC (and all other predictors/covariates) with the experimental condition on anger are presented in Table 3. Since we showed an increase in anger after failure and this paper investigates responses to failure, in the following only anger after failure will be further analyzed.

**Table 3:** Covariates in mixed model on anger in interaction with point of measurement

Measure	F(1.39, 261.55)	*p* ^b^	partial η2
NFC^a^	14.76	< 0.001	0.073
PSE^a^	0.73	0.436	0.004
Trait anger^a^	32.00	< 0.001	0.145
Age^a^	31.57	< 0.001	0.144
Gender^a^	4.85	0.018	0.025

*Note. N* = 194. Mean scores of anger after working on the anagram and tangram tasks were used. ^a^in interaction with point of measurement ^b^Greenhouse-Geisser correction (*Mauchly-W*(2) = 0.562, *p* < .001)

### PSE as moderator of the relationship between NFC and anger after failure

**Fig. 3:**
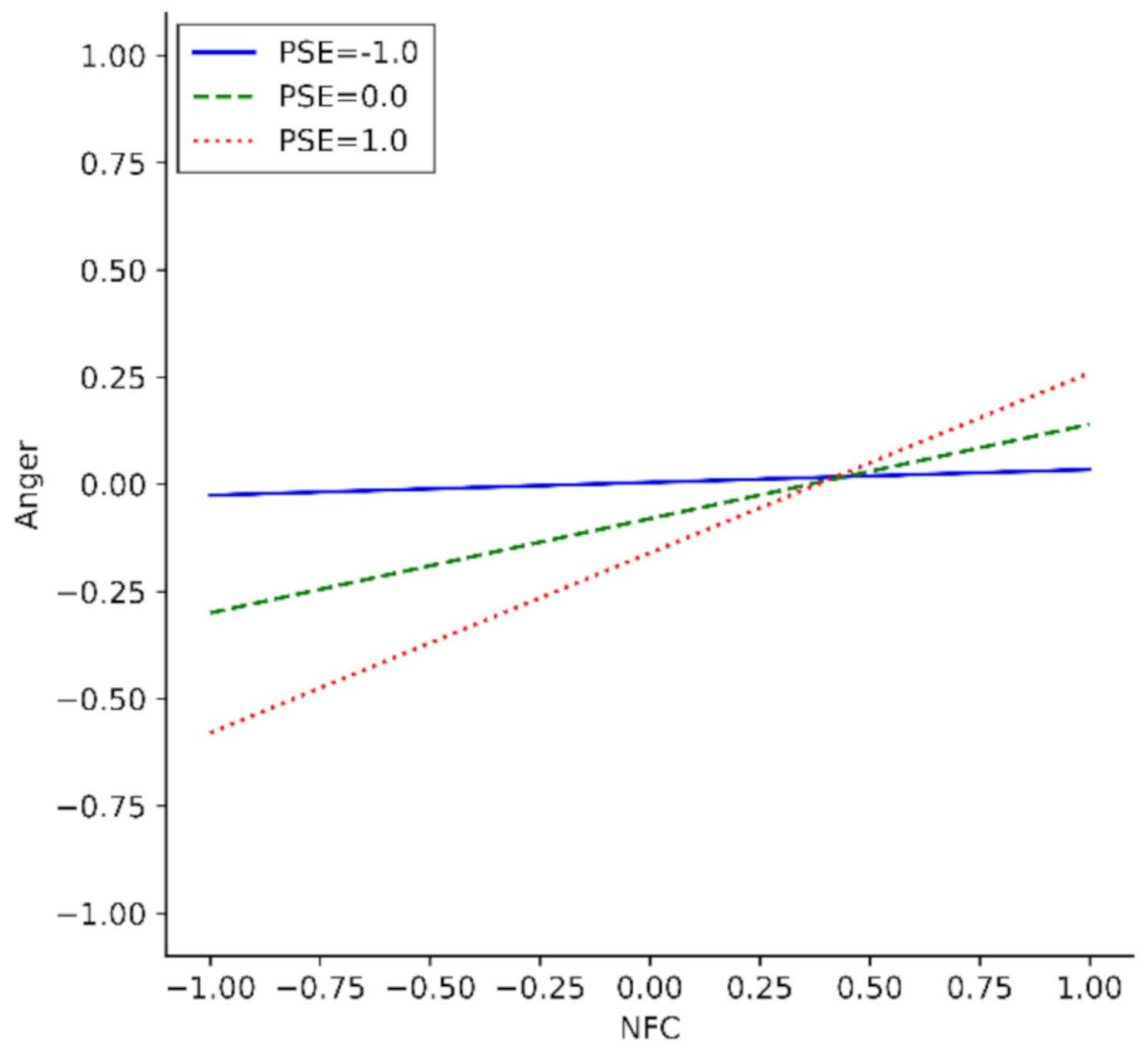
Moderation Analysis: Moderating effect of PSE on the effect of NFC on Anger after Failure Note. *N* = 194. Overall Model: *F*(3,190) = 6.20, *R*^*2*^ = 0.07, *p* < .001. Mean scores of anger after working on the unsolvable anagram and tangram tasks were used

A moderation-analysis examining whether PSE moderates the effect of NFC on anger after failure with heteroscedasticity-consistent standard errors (HC3 by Davidson & MacKinnon) and 5000 bootstrap samples (overall model: *F*(3,190) = 6.20, *R*^*2*^ = 0.07, *p* < .001) revealed an effect of the interaction of NFC × PSE on anger after failure (*b* = 0.19, with *ΔR*^*2*^ = 0.05, *p* = .027, bootstrapped 95% CI [0.03, 0.33]). Z-scores were used for better interpretability. When PSE was lower (-1) there was no relationship between NFC and anger (*b* = 0.03, 95% CI [-0.17, 0.22], *t* = 0.25, *p* = .801). There was a relationship between NFC and anger when PSE was moderate (*0*) (*b* = 0.22, 95% CI [0.11, 0.34], *t* = 3.79, *p* < .001) or higher (1) (*b* = 0.42, 95% CI [0.20, 0.64], *t* = 3.74, *p* < .001). Thereby, the higher an individual’s PSE was, the more did their NFC affect their anger after failure (Fig. 3). Further, the moderation analysis showed no direct effect of PSE on anger after failure (*b* = − 0.08, 95% CI [-0.23, 0.07], *t* = -1.07, *p* = .287) but - consistent to the results of the mixed model analysis - a positive direct effect of NFC on anger after failure (*b* = 0.22, 95% CI [0.10, 0.34], *t* = 3.76, *p* < .001).

### Effects of NFC, PSE, and anger on the probability of intented persistence

Multinomial logistic regression was applied to compare between individuals with no intention to persist (*n* = 62), intention to persist after one task (*n* = 46), and intention to persist after both failed tasks (*n* = 86). Z-Scores were used for better comparability of effects. Not wanting to try again after both tasks (no intention to persist) was used as reference category. Bootstrapping with 5000 samples was applied. To analyze the effect of anger on persistence after failure, we included NFC, PSE, anger after failure, the interaction of NFC and anger, the interaction of PSE and anger and the interaction of NFC, PSE, and anger into our regression model. The effect size of the final model (*χ*^*2*^ = 98.06, *df* = 12, *p* < .001, *n* = 194) was medium (*R*_Nagelkerke_ = 0.45). Results showed that PSE, NFC, the interaction of PSE and anger, and the interaction of PSE, NFC and anger had significant positive effects on the probability of the intention to persist after both tasks, while only NFC positively affected the probability of intending to persist after only one task (Table 4).

**Table 4:** Multinomial logistic regression: effects on persistence after failure

Persistence	Predictor	Exp(B)	Wald z^2^	*p*	95% CI	
					LL	UL
wanting to try again after only one task (1)	NFC	3.78	13.63	< 0.001	1.87	7.66
	PSE	1.55	2.81	0.094	0.93	2.57
	Anger	1.10	0.05	0.816	0.49	2.46
	NFC*Anger	1.39	0.36	0.548	0.47	4.09
	PSE*Anger	1.58	1.39	0.238	0.74	3.38
	PSE*NFC*Anger	1.32	0.49	0.484	0.61	2.87
wanting to try again after both tasks (2)	NFC	2.77	7.33	0.007	1.33	5.80
	PSE	3.84	11.96	< 0.001	1.79	8.23
	Anger	1.18	0.18	0.675	0.55	2.54
	NFC*Anger	1.13	0.04	0.846	0.34	3.77
	PSE*Anger	7.82	8.70	0.003	2.00	30.69
	PSE*NFC*Anger	2.58	8.17	0.004	1.35	4.93

*Note*. *N* = 194. *R*^2^_Nagelkerkes_ = 0.45. Reference category: “not wanting to try again at all” (0) (*n* = 62). Mean scores of anger after working on the anagram and tangram tasks were used

The following regression equation was used to visualize the effects of anger after failure (x_1_) in interaction with PSE (x_2_) and NFC (x_3_) on the probability of intended persistence after both tasks. The range of z-standardized variables in the equation was − 1 to + 1.

$$\displaylines{ p = f\left( {{x_1},{x_2},{x_3}} \right) = \cr \frac{1}{{1 + {e^{ - \left( {0.567 - 0.164{x_1} + 1.346{x_2} + 1.020{x_3} + 2.057{x_1}{x_2} + 0.120{x_1}{x_3} + 0.947{x_1}{x_2}{x_3}} \right)}}}} \cr} $$

**Fig. 4:**
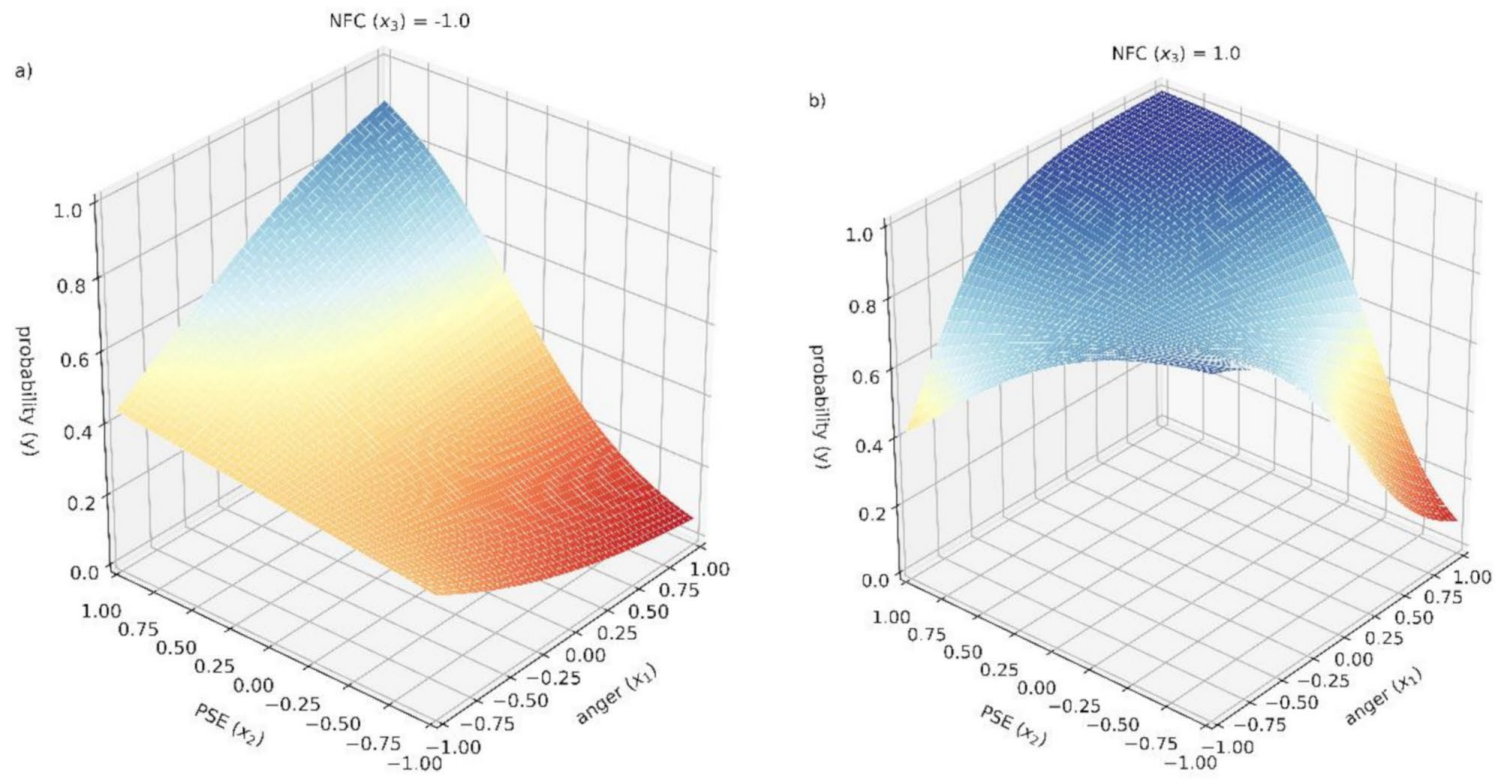
Multinomial Logistic Regression: Effect of PSE x NFC x Anger on the probability of showing persistence after two tasks^a^ Note. *N* = 194. *R*^2^_Nagelkerkes_ = 0.45. Reference category: “not wanting to try again at all”. Mean scores of anger after working on the anagram and tangram tasks were used. ^a^wanting to try solving both anagram and tangram again after failure

The direct positive effect of NFC on the probability to persist (y axis) can be observed in the greater blue area comparing greater NFC (Fig. 4b) with lower NFC (Fig. 4a). Furthermore, individuals with higher NFC have a higher probability to show intention to persist even when anger and PSE are low (Fig. 4b). Moreover, the direct positive effect of PSE (x_2_-axis) can be observed in the graph going upwards towards the back and with that in showing a greater blue area towards the back in both Fig. 4a and b. Anger (x_1_-axis) does not show any direct effect on the probability of intention to persist. Further, the interaction effect of anger and PSE can be observed as such as individuals high in anger and PSE have a greater probability to show persistence in general. Additionally, individuals high in anger but low in PSE have a lower probability to show intention to persist after failure independently of their NFC. Regarding the three-way interaction of anger, NFC, and PSE, it can be observed that the probability to persist is highest when NFC is high and also PSE as well as anger are greater than the mean (Fig. 4b), while the probability to persist is rather low if anger, PSE and NFC are lower (Fig. 4a).

## Discussion

In this study, we expected NFC to be positively related to anger as an emotional response to failure in cognitively challenging tasks. We further expected that PSE would moderate this effect of NFC on anger and that anger, NFC, and PSE would affect the intention to persist on the task after failure. Our results support these predictions, with anger affecting persistence only in interaction with PSE and NFC.

### NFC and the emotional response to failure

Results of the mixed model suggest that individuals show greater anger after failure compared to baseline, with no difference between anger at baseline and anger after success. These results support many previous findings that describe anger as a response to failure [1, 2, 7–11].

Thereby, the results indicate that at baseline and after success the variance of anger scores was low. However, the observed higher variance after failure suggests that participants showed different responses depending on their personality pattern. In line with that, the mixed models also showed that in response to failure, higher-NFC individuals showed significantly higher levels of anger than those lower in NFC. Thus, NFC had a specific effect on anger in response to failure whereas it had no direct effect on general anger. This pattern suggests that although individuals with higher NFC generally show less negative emotionality, as supported by various studies [41, 48, 71], they appear to be more negatively affected by failures in cognitively demanding tasks. In addition, the fact that individuals with higher NFC scores showed more anger after a failure than at baseline or after success may support previous findings suggesting that individuals with higher NFC are not only motivated to solve effortful cognitive tasks, but are also motivated to avoid failure and an incompetent self-image [18, 19, 34]. By that, previous studies have shown a positive correlation between anger and needs of the ego [3, 36].

### The moderating role of PSE

The moderation analysis showed that PSE moderated the effect of NFC on anger in response to failure. That is, NFC predicted anger only if individuals were medium to high in PSE. This suggests that the more an individual believes in their own competence, the more does cognitive motivation influence the emotional response to failure. These findings are in line with the conceptual view of PSE as the expectation of accomplishing a task by one’s own abilities [22, 24] and with the assumption that failure might only be experienced if expectations were generated [5]. Thus, if an individual believes to be able to solve complex tasks in general (moderate to high PSE) and furthermore, invests greater effort to solve a cognitively demanding task (high NFC), failures may be less expected and may therefore lead to greater anger. Moreover, PSE builds on the integration of experiences of failure and success into the self [20]. Thereby, individuals higher in NFC positively evaluate experiences more often and have better long-term integration of such experiences [48, 72]. Therefore, individuals high in PSE and NFC might have been more confident to solve a task, which might have led to greater anger after failure [39]. In addition, Stone [23] showed that individuals with higher PSE tend to rate their performance in cognitively complex tasks higher than it actually is and therefore, they might be more frustrated after failing if NFC is high.

Moreover, we expected that anger would be lower in individuals with lower NFC relatively independent of their PSE, as individuals with lower NFC have been shown to be hardly influenced by expectations of their performance [34]. However, results in the present study suggest that anger in lower-NFC individuals is lower in individuals with medium to high PSE while anger is moderate when PSE is low. By that, individuals lower in PSE showed rather high levels of anger regardless of their NFC. Therefore, individuals lower in PSE might feel confirmed in their expectation of not being able to solve a task and hence, feel anger and frustration after failure in general. In contrast, individuals who are high or medium in PSE but lower in NFC may reason their lower motivation and lower effort to be responsible for failing instead of reasoning a lack of ability [21]. This argumentation would protect their ego and therefore, may not lead to anger. As a result, we presume that an individual’s intrinsic cognitive motivation will only affect their emotional response to failure if they have positive expectations regarding their own abilities.

### The behavioral response to failure

Furthermore, we addressed the question whether greater anger would motivate higher-NFC individuals to try to solve a task again, that is, to show persistence behavior. By that, we assessed the intention to persist, whereby intention is reported to reflect the extent of which an individual has thought through the consequences of their decision of action [55]. As expected, NFC was positively associated with the intention to persist after failure. Hence, our results substantiate the findings of Fleischhauer et al. [41] and Reinhard and Dickhäuser [22], who reported that individuals high in NFC described themselves as being more persistent. Similar to the results on NFC, our results on PSE were in line with previous findings indicating that PSE is positively related to persistence [5, 20, 25]. Anger after failure itself did not affect the probability of intention to persist directly. However, our results indicate that there is a significant effect of anger in interaction with PSE and NFC. In line with the moderation analyses on anger after failure, where anger mostly occurred in individuals high in NFC and PSE, there was a three-way interaction of NFC, PSE, and anger on persistence. Taken together, the results suggest that anger will positively affect the probability of intention to persist if an individual’s NFC and PSE are medium to high. This pattern of results might indicate that individuals with higher NFC and PSE show greater anger after failure (see mixed model analysis) and further, may use anger as an impulse for showing persistence. In line with our findings, Schmitt et al. [40] reported that anger might lead to greater persistence when individuals are more committed. Hence, anger can function as an activating affect [4, 7, 36, 40], but only in interaction with certain personality traits.

In contrast, anger after failure that is not associated with higher NFC and PSE might result from a feeling of possessing a lack of ability, which may lead to the need to escape the situation rather than the need to persist [9, 37]. This goes in line with our results showing that if PSE is low, higher anger levels lead to a very low probability to intent to persist in general. Indeed, previous literature stated that anger may only functions as an activating emotion if it enhances the feeling of being in control [4, 6, 39].

Interestingly, NFC might positively affect persistence even if PSE and anger are lower. Here, lower anger might be considered an indicator for not experiencing failure despite not being able to solve the tasks. The higher probability of persistence behavior might support previous findings of Britt et al. [73] reporting that the more engaged individuals are in working on a task the more likely they are to try to achieve their goal of solving it. Our results are also in line with the research of Arrington who showed that individuals higher in NFC are better able to adapt to a situation after failure [46].

In sum, results of the multinomial logistic regression model suggest that NFC and PSE affect the probability of intention to persist positively in general, whereas anger affects the intention to persist differently depending on an individual’s NFC and PSE.

### Limitations and future directions

Although the study has many strengths such as the use of an experimental approach to examine effects of NFC on the emotional response after failure and on the behavioral consequences of anger in this context, as well as a rather large sample size and sufficient power to detect small effects, several limitations have to be discussed. Our study examined the consequences of failure in cognitively demanding tasks. Two tasks – a figural (tangram) and a verbal (anagram) task - were used. Future research should apply other tasks to generalize and replicate the effects found in the present study.

Additionally, the sample must be considered biased due to the recruitment strategy (i.e. non-probabilistic sampling methods) and the disproportionate representation of younger (median age = 28 years) and female participants (57%). While not representative of the general population, the sample displays greater demographic heterogeneity than is typically observed in psychological studies, that often rely on undergraduate student samples. Nevertheless, the generalizability of the findings is limited. Future research should aim to replicate these results in larger and more demographically diverse samples to strengthen external validity and further test the robustness of the reported effects.

Furthermore, anger was measured using the STAXI [52], which contains items for rather high degrees of anger that are rather difficult to agree with, resulting in overall lower anger scores. This might have limited the size of effects. Future research may use measures that better differentiate lower levels of anger. Besides anger, other affects and their association with persistence (including deactivating affects such as boredom) could be assessed. Moreover, besides PSE, other moderators or mediators of the relationship between NFC and emotional and behavioural responses to failure should be examined. For example, self-control might be an interesting candidate, as it is positively linked to NFC [47, 74] and has been shown to mediate the association of NFC and affective states [75]. Additionally, it may correlate with greater persistence despite negative feedback and thus explain the effect of NFC on persistence behaviour. In addition to questionnaires, more objective biological measures could be used as criterion, such as cortisol measurements that indicate individuals’ stress responses to failure in cognitively challenging situations. Moreover, a measure of feeling to have failed could be included in future studies to further validate our findings.

The findings of this study might have important implications, especially in the educational context, where the effects of NFC have been well-documented [27, 42, 43] and where responses to failure and their impact on persistence can significantly affect an individual’s academic trajectory. Specifically, understanding how individuals with high NFC and high PSE respond emotionally and behaviourally to failure can inform strategies to promote persistence. For individuals with high NFC and high PSE, anger in response to failure can actually serve as a motivator, encouraging them to keep trying. Educators could capitalize on this by providing opportunities for students to reattempt tasks after failure, turning their emotional response into a drive for continued effort. Additionally, enhancing students’ self-efficacy could help them better cope with failure and channel anger into motivation, ultimately fostering greater persistence in challenging tasks. Interventions aimed at boosting PSE may help students persevere, even in the face of setbacks. Furthermore, greater insight into how anger can be used positively as an activating emotion and how to learn from failure [46], could help reduce dropout intentions in both academic and work contexts. While this study assessed the intention to persist after failure, future studies should also examine actual persistence behaviour after failure.

## Conclusions

The present study investigated whether intrinsic motivation to solve cognitively demanding tasks (NFC) affects responses to failure in such tasks. Our results support previous findings presuming anger to be an emotional response to failure. Further, our results suggest that NFC affects anger after failure and that this effect is moderated by PSE. Hence, only if individuals believe in their ability (medium to high PSE), the motivation to work on a cognitively demanding task (NFC) will positively affect anger. Moreover, the results imply that anger as an emotional response to failure affects the probability of intention to persist in individuals higher in PSE and NFC. Our data suggests that the effects of failure on emotions and the effect of emotions on behavioral responses reported in previous studies may depend on certain personality traits such as NFC and self-beliefs such as PSE. Finally, we conclude that failure can affect an individual’s emotions and their behavior differently depending on their personality characteristics and self-beliefs pointing out that individuals with different personality traits might need different support in the event of failure.

## Data Availability

The datasets generated and analyzed in the current study, the syntax, and the dataset legend as well as the tangram tasks are available in the figshare repository, DOI’s: 10.6084/m9.figshare.14892606, 10.6084/m9.figshare.14892816, 10.6084/m9.figshare.14892786, and 10.6084/m9.figshare.29365304.v1. Anagrams used were negrom (solved: Morgen) and reknkeusnha (unsolvable).
